# Efficacy of Donated Milk in Early Nutrition of Preterm Infants: A Meta-Analysis

**DOI:** 10.3390/nu14091724

**Published:** 2022-04-21

**Authors:** Yu Li, Cheng Chi, Cheng Li, Junyan Song, Zanmin Song, Wenjun Wang, Jing Sun

**Affiliations:** 1 School of Nursing, Weifang Medical University, Weifang 261042, China; ly992276043@163.com; 2 School of Nursing, Jining Medical University, Jining 272067, China; chic@mail.jnmc.edu.cn (C.C.); lc454419096@163.com (C.L.); 13791798026@126.com (J.S.); 3 School of Medicine and Dentistry, Griffith University, Gold Coast, QLD 4215, Australia; z.song@griffith.edu.au; 4 Menzies Health Institute Queensland, Griffith University, Gold Coast, QLD 4215, Australia

**Keywords:** donated milk, infant formula, prematurity, very low birth weight

## Abstract

Background: Preterm birth is associated with an increased risk of many complications, which is a main public health problem worldwide with social and economic consequences. Human milk from breast feeding has been proved to be the optimal nutrition strategy for preterm infants when available. However, the lack of human milk from mothers makes formula widely used in clinical practice. In recent years, donated breast milk has gained popularity as an alternative choice which can provide human milk oligosaccharides and other bioactive substances. Objective: We aimed to conduct a systematic review and meta-analysis to evaluate the nutritional effects of donated breast milk on preterm infants compared with formula. Method: In the present study, we searched Medline, Web of Science, Embase, clinicaltrials.gov, the China national knowledge infrastructure, and the Cochrane central register of controlled trials for candidate randomized controlled trials (RCTs). Results: A total of 1390 patients were enrolled in 11 RCTs and meta-analysis results showed that donated breast milk is also more advantageous in reducing the incidence of necrotizing enterocolitis (NEC, RR = 0.67, 95% CI = 0.48 to 0.93, *p* = 0.02), reducing the duration of parenteral nutrition (MD = −2.39, 95% CI = −3.66 to −1.13, *p* = 0.0002) and the time of full enteral feeding (MD = −0.33, 95% CI = −3.23 to 2.57, *p* = 0.0002). In comparison, formula significantly promotes the growth of premature infants, including their weight gain (MD = −3.45, 95% CI = −3.68 to −3.21, *p* < 0.00001), head growth (MD = −0.07, 95% CI = −0.08 to −0.06, *p <* 0.00001) and body length (MD = −0.13, 95% CI = −0.15 to −0.11, *p* < 0.00001), and reduces the time it takes for premature infants to regain birth weight (MD = 6.60, 95% CI = 6.11 to 7.08, *p* < 0.00001. Conclusion: Compared with formula, donated breast milk could significantly reduce the incidence of NEC, the duration of parenteral nutrition, and the time of full enteral feeding. Adding fortifiers in donated milk could make it as effective as formula in promoting the physical growth of premature infants.

## 1. Introduction

Premature infants are defined as infants born at less than 37 weeks of gestation. Many studies have shown that prematurity often leads to postnatal stunting and increased morbidity and mortality due to immature organ development [1]. This worldwide public health problem creates a social and economic burden. Premature infants cannot absorb the required nutrients properly due to gastrointestinal insufficiency [2], which causes the slow growth and high morbidity of these infants. Adequate nutrition is known to play an important role in premature infants [3]. Currently, the nutritional supply comes from three major sources: breast feeding, formula, and donated breast milk. Breastfeeding has been the mainstay for early nutrition of premature infants, since it not only meets the nutritional needs of premature infants, but also provides antibodies for weak premature infants to enhance their immunity [4]. When the mother of a premature infant does not produce sufficient breast milk, however, formula becomes a common fallback in order to rectify the nutritional deficiency of premature infants in clinical practice [5]. In recent years, donated breast milk, which is pasteurized and stored in milk banks, has gained popularity in clinical practice due to several mechanisms. Firstly, donated breast milk retains much of its nutrients and has advantage over formula in terms of physical development, because formula has more protein and fat contents [6]. Secondly, donated breast milk has the advantages over formula in immune function, even though pasteurization does destroy part of the immune components [7]. Thirdly, donated breast milk retains the oligosaccharide content of fresh breast milk, which regulates immune cells and reduces the incidence of infection [8], including necrotizing enterocolitis (NEC) and sepsis in preterm infants. The specific mechanism is not clear, but it has been hypothesized that breast milk contains bioactive substances that reduce NEC and sepsis, whereas formula contains risk factors that increase NEC [9]. More specifically, breast milk contains bioactive compounds, most notably antioxidants, which counteracts the effects of oxidative stress in newborns. Donated breast milk has less bioactive factors over time, but is still superior to formula [10]. The increased oxidative stress in preterm infants aggravates the perinatal morbidity of these infants [11], such that donated breast milk may reduce premature mortality and hospital stay.

Donated breast milk, rather than formula, has been recommended by many countries as a substitute for breast feeding. The full advantages of donated breast milk should be re-evaluated, in spite of the known benefits of formula to the physical development of premature infants. The only few trials that compared the use of donated breast milk and formula in premature infants produced inconsistent results. Data from Yu et al. [12] showed that formula was significantly superior to donated breast milk in terms of weight gain and body length growth in preterm infants [6]. The few published meta-analyses also reached consensus. For instance, inconsistent results have shown that formula was found to be significantly better than donated breast milk in promoting the growth of head circumferences in one study [6], but was not in another study [12]. To further evaluate the nutritional effects of donated breast milk on multiple outcome indicators in premature infants, we included more randomized clinical trials (RCTs) in outcome indicators including the reduction of incidence of NEC, sepsis, length of hospital stay, mortality, the time of parenteral nutrition, and the time of full enteral feeding and physical growth. Using large data and extensive variables, this study provides the latest evidence for the clinical use of donated breast milk.

## 2. Materials and Methods

This systematic review and meta-analysis was designed in line with the criteria suggested by the Cochrane collaboration. We used the preferred items for systematic review and meta-analysis (PRISMA) criteria to guide the reporting of the results [13]. As this study was based on published data, the permissible consent from participants was not applicable. The meta-analysis was registered with the PROSPERO database (registration number CRD42022308958).

### 2.1. Literature Search Strategy

We conducted a systematic search of the literature to identify all randomized controlled trials that involved the intervention in premature or low birth weight infants with donated breast milk. We searched Web of Science (from 1946 to November 2021), PubMed (from 1966 to November 2021), Embase (from 1974 to November 2021), China National Knowledge Infrastructure (from 1976 to November 2021), clinicaltrials.gov and the Cochrane Central Register of Controlled Trials (from 1997 to November 2019) to identify potentially eligible studies with no language restriction. Details of search terms and strategies are included in Supplemental Figure S1.

### 2.2. Inclusion and Exclusion Criteria

We selected RCTs that enrolled preterm (gestational age less than 37 weeks) or low birth weight (birth weight less than 2500 g) infants as participants. In these RCTs included, donated breast milk was used in the experimental group and formula was used in the control group. At least one of the following outcome indicators was included: incidence of NEC, length of hospital stay, mortality, sepsis, and duration of parenteral nutrition, and retinopahty of prematurity. In addition, weight gain, health growth, linear growth, time to regain birth weight and time to full enteral feeding were included. We excluded conference proceedings and abstracts without full RCTs information, as well as some studies with incomplete data. For duplicates of published studies, we selected the article with the most complete data and most recent results.

### 2.3. Date Extraction

In this study, two researchers screened through the literature independently and completed the extraction of data. The extracted results were then a cross-checked. Disagreements were resolved through discussion and judged by a third investigator. For literature that lacked original data, we tried to get in touch with the authors to obtain the raw data, otherwise, the studies were excluded. The extracted information includes the following aspects: first author’s name, year of publication, country, sample size, details of the intervention, gestational age, gender, birth weight, key elements of the bias risk assessment, and the data of outcome indicators.

### 2.4. Quality and Risk of Bias Assessment

The quality of all included studies was assessed by the JADAD scale, which evaluated four aspects including randomized sequence generation, randomized concealment, blinding, and withdrawal. The full score for this tool was 7, and we considered 1 to 3 as low-quality literature and 4 to 7 as high-quality literature. We used the Cochrane risk of bias tool for RCTs to evaluate the risk of bias of included literature, which included seven evaluation items: sequence generation, allocation concealment, blinding of participants, outcome evaluator, incomplete outcome data, and selective outcome reporting. The included literature was evaluated as low risk, high risk or unclear. This assessment was completed by two evaluators independently. When disagreement arose, it was resolved through negotiation with a third researcher.

### 2.5. Outcomes

A total of 11 outcome indicators were meta-analyzed through literature integration. The primary outcome indicators included incidence of NEC, length of hospital stay, mortality, sepsis, parenteral nutrition time, and incidence of retinopathy of prematurity. Secondary outcome indicators included the growth and development of premature infants, such as daily weight gain, weekly growth of head circumference and body length, time to regain birth weight, and time to full enteral feeding.

### 2.6. Statistical Analysis

The data was analyzed by Revman 5.3 software (Version 5.3, Copenhagen: The Nordic Cochrane Centre, The Cochrane Collaboration, Denmark). The results of dichotomous data were presented as risk ratio (RR) and 95% confidence interval (CI), while the results of continuous data were presented as mean difference (MD) and 95% CI. The χ^2^ and *I*^2^ tests were used to evaluate the heterogeneity of studies. A sensitivity analysis referred to the re-meta-analysis of data after sequentially removing single studies, and compared the results after elimination with the original results. Subgroup analysis was performed to identify the effects of birth weight, fortifier, publication date, country, number of participants, and number of experimental selection centers. In four articles [14,15,16,17], the data was expressed in the form of the median, therefore we used the algorithm of Hozo et al. [18] to estimate the weighted mean and standard deviation. Similarly, the test for overall effects determined the result by the *p*-value magnitude, i.e., when *p* < 0.05 the data was considered to be statistically significant. Publication bias was determined by funnel plots.

## 3. Results

**Figure 1:**
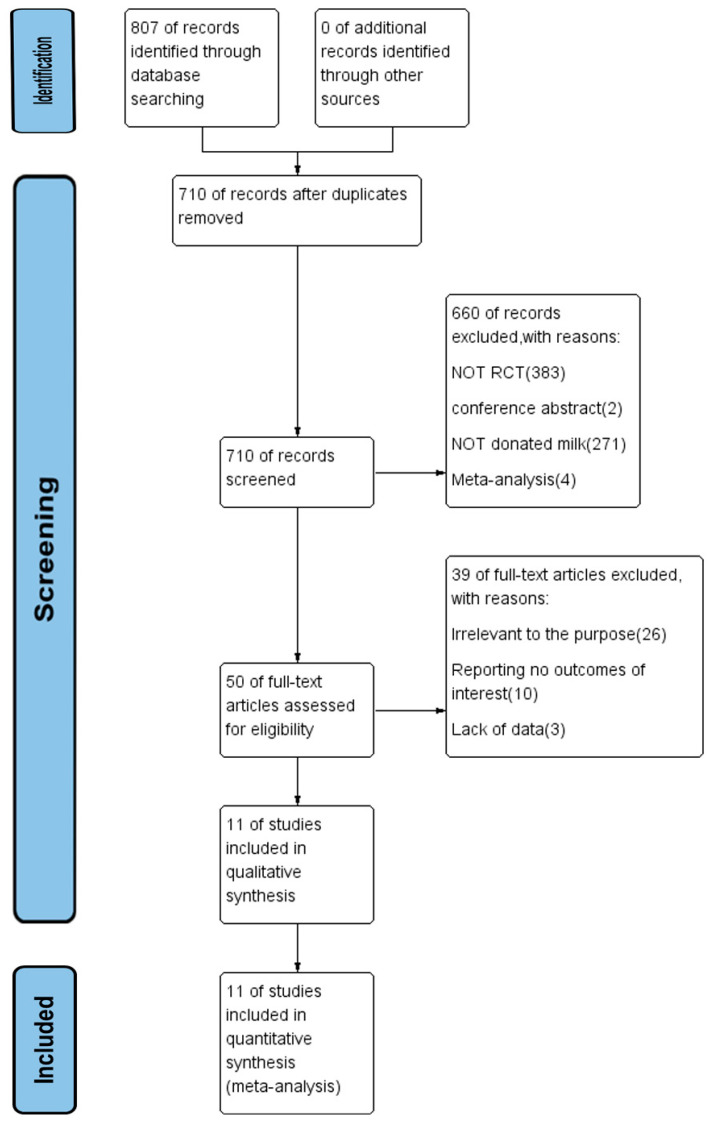
Flow chart of the search strategy and search results. The relevant number of papers at each point is given. RCT refers to randomized controlled trial.

The screening process of RCTs for meta-analysis was presented in Figure 1. After an initial systematic search, a total of 807 articles were retrieved, and 710 articles remained after the removal of duplicate articles. Next, the title and abstract of the articles were screened. The excluded papers consisted of 383 non RCTs, two conference abstracts and four meta-analyses papers. A further 271 papers did not use donated breast milk as the intervention group and hence were excluded. We screened the full text of the remaining 50 papers, and excluded a further 26 papers with irrelevant research objectives, 10 papers without the outcome indicators that we needed, and three papers with incomplete data. Finally, 11 papers were included for the current study.

### 3.1. Characteristics of Included Studies

Table 1 describes the detailed characteristics of the included literature. These papers were published between 1977 [19] and 2018 [20]. Five studies were conducted in European countries [16,19,20,21,22], five in the United States [14,15,23,24,25] and one in Canada [17]. A total of 1390 patients were included in our study, among which the study by Corpeleijn et al. [21] included 373 patients (the highest number of cases), while Putet et al. [22] included 12 patients (the lowest number of cases). The birth weight of premature infants among the 11 papers was distributed as follows [14,15,16,17,19,20,21,22,23,24,25]: not reported (both the gestational age and birth weight) in 1 study [25]; less than 1000 g in four studies [14,15,17,23]; more than 1500 g in one study [19]; between 1000 g and 1500 g in all the remaining studies [16,20,21,22,24]. Fortifiers were added to donated breast milk in four studies [14,17,21,23]. The sterilization method for donated breast milk was not described in one study [16], whereas the milk used in other studies [14,15,17,19,20,21,22,23,24,25] was pasteurized.

### 3.2. Primary Outcomes

#### 3.2.1. Incidence of NEC

**Figure 2:**
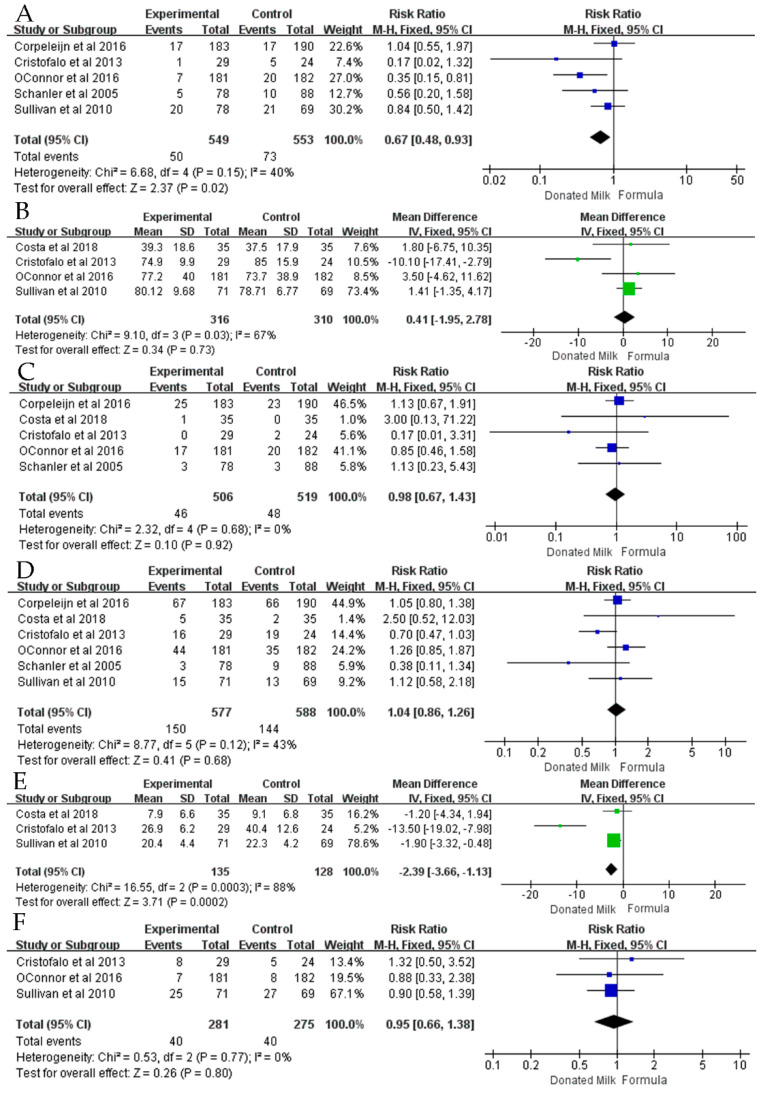
The forest plot shows primary outcomes. (**A**) Incidence of NEC (**B**) Length of Hospital Stay (**C**) Mortality (**D**) Incidence of sepsis (**E**) Duration of parenteral nutrition (**F**) Incidence of retinopathy of prematurity [14,15,17,20,21,23].

Five studies [14,15,17,21,23] reported the incidence of NEC (Figure 2A), and meta-analysis showed that donated breast milk significantly reduced the incidence of NEC (RR = 0.67, 95% CI = 0.48 to 0.93, *p* = 0.02) and that the heterogeneity is low (*I*^2^ = 40%, *p* = 0.15). Subgroup analysis was performed according to whether the trial was single-center or multi-center. The results (Table 2, Figure 2A) show that donated breast milk significantly reduced the incidence of NEC in multi-center trials, and there was no significant difference between donated breast milk and formula in single-center trials.

#### 3.2.2. Length of Hospital Stay

Four articles [14,15,17,20] reported the length of hospital stay (Figure 2B), and meta-analysis showed that neither donated breast milk nor formula significantly reduced the length of hospital stay (MD = 0.41, 95% CI = −1.95 to 2.78, *p* = 0.73), and the heterogeneity was very large (*I*^2^ = 67%, *p* = 0.03). Subgroup analysis was conducted according to whether fortifiers were used in the trial or according to the number of participants in the trial. A subgroup analysis showed that neither donated milk nor formula significantly reduced the length of hospital stay for preterm infants. Heterogeneity was significantly reduced in the fortifier group and in the cohort with more than 100 participants, as detailed in Table 2, Figure 2B.

#### 3.2.3. Mortality

Five articles [14,17,20,21,23] reported the mortality of premature infants (Figure 2C), and meta-analysis results showed that no significant difference could be observed between donated breast milk and formula in reducing the mortality of premature infants (RR = 0.98, 95% CI = 0.67 to 1.43, *p* = 0.92) and the heterogeneity was low (*I*^2^ = 0%, *p* = 0.84). Subgroup analysis was performed based on the number of centers used, country, birth weight of preterm infants, and number of trials included in the study. There was no significant difference between donated milk and formula after grouping, as detailed in Table 2, Figure 2C.

#### 3.2.4. Incidence of Sepsis

Six articles [14,15,17,20,21,23] reported the incidence of sepsis in preterm infants (Figure 2D). There was no significant difference between the two types of nutrition strategies in reducing the incidence of sepsis (RR = 1.04, 95% CI = 0.86 to 1.26, *p* = 0.68) with low heterogeneity (*I*^2^ = 43%, *p* = 0.12). We performed subgroup analyses based on whether breast milk was fortified, the number of centers, country, birth weight, and number of enrolled patients. There was no significant difference between donated milk and formula after grouping, as detailed in Table 2, Figure 2D.

#### 3.2.5. Duration of Parenteral Nutrition

Three articles [14,15,20] reported the duration of parenteral nutrition for premature infants, and the results of the meta-analysis showed that donated breast milk significantly reduced the duration of parenteral nutrition for premature infants (MD = −2.39, 95% CI = −3.66 to −1.13, *p* = 0.0002), as detailed in Figure 2E.. Heterogeneity was significantly reduced after one article [14] was removed from the sensitivity analysis (*I*^2^ = 0%, *p* = 0.69).

#### 3.2.6. Incidence of Retinopathy of Prematurity

The incidence of retinopathy of prematurity was reported in three articles [14,15,17]. The meta-analysis results showed that donated breast milk was more beneficial in reducing the incidence of retinopathy of prematurity (RR = 0.95, 95% CI = 0.66 to 1.38, *p* = 0.70), although there was no statistically significant difference and low heterogeneity (*I*^2^ = 0%, *p* = 0.77), as detailed in Figure 2F.

### 3.3. The Secondary Outcome

#### 3.3.1. Weight Gain

**Figure 3:**
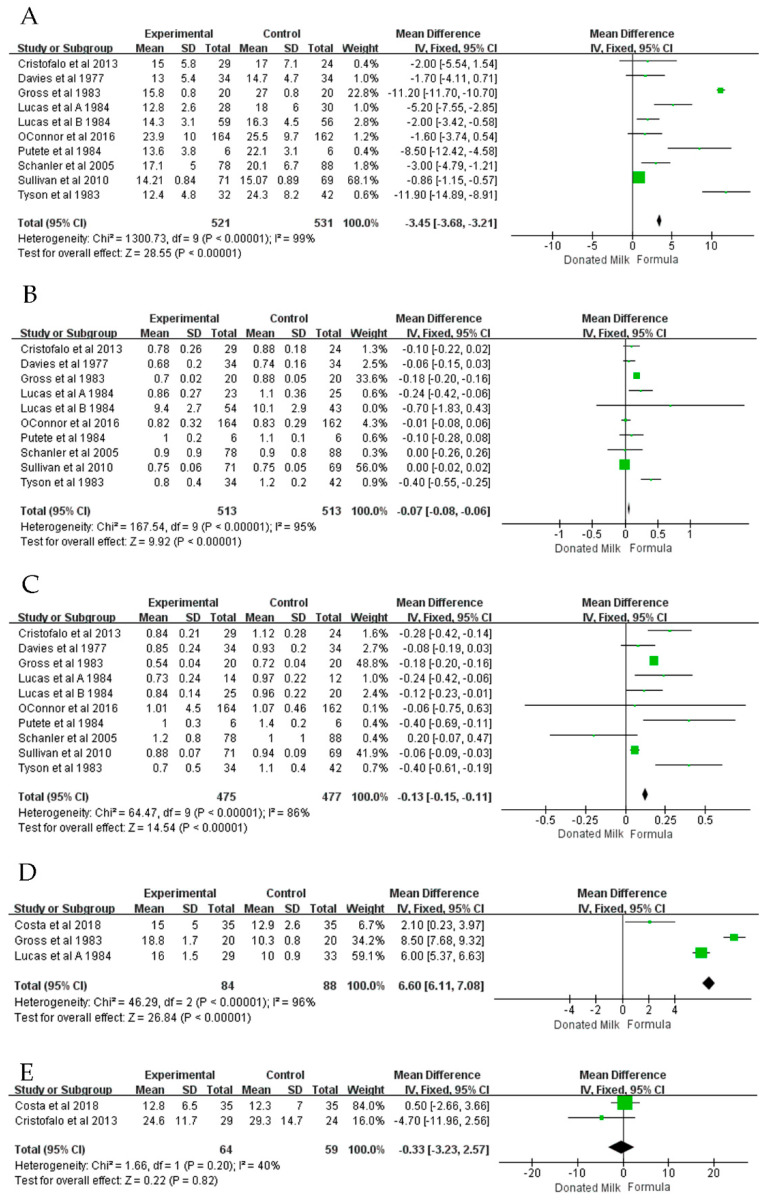
The forest plot shows secondary outcomes. (**A**) Weight gain (**B**) Head growth (**C**) Linear growth (**D**) Time to regain birth weight (**E**) Time to full enteral feeding [14,15,16,17,19,20,22,23,24,25].

Nine articles [14,15,16,17,19,22,23,24,25] reported daily weight gain in premature infants (Figure 3A), and a meta-analysis showed that formula had a significant advantage over donated breast milk in the weight gain of premature infants (MD = −3.45, 95% CI = −3.68 to −3.21, *p* < 0.00001) and also showed high heterogeneity (*I*^2^ = 99%, *p* < 0.00001). We performed a subgroup analysis based on whether the trial used fortifier, birth weight, publication date, country, number of centers, and number of participants. The results of the subgroup analysis showed that after grouping, formula always had a significant advantage in the weight gain of premature infants, as detailed in Table 3.

#### 3.3.2. Head Growth

Nine articles [14,15,16,17,19,22,23,24,25] reported weekly growth in head circumference for premature infants (Figure 3B), and meta-analysis results showed that formula had a significant advantage over donated breast milk in head circumference growth (MD = −0.07, 95% CI = −0.08 to −0.06, *p* < 0.00001), and the heterogeneity was high (*I*^2^ = 80%, *p* < 0.00001). Subsequently, we performed a subgroup analysis based on donated milk with fortifier, birth weight, publication date, country, number of centers, and number of participants. A subgroup analysis (Table 3) showed that donated breast milk was not inferior to formula in infants less than 1000 g in weight when fortifiers were added.

#### 3.3.3. Body Length Growth

Nine articles [14,15,16,17,19,22,23,24,25] reported the effects of donated breast milk and formula on weekly body length gains in premature or low birth weight infants (Figure 3C). Meta-analysis results showed that formula had a significant advantage over donated breast milk in terms of body length (MD = −0.13, 95% CI = −0.15 to −0.11, *p* < 0.00001), but the heterogeneity was high (*I*^2^ = 86%, *p* < 0.00001). We performed a subgroup analysis based on the use of fortifiers in donated breast milk, the birth weight of preterm infants, publication date, country, number of centers, and number of participants. The results of the subgroup analysis showed that after grouping, formula always had a significant advantage in the linear growth of premature infants, as detailed in Table 4.

#### 3.3.4. Time to Regain Birth Weight

Three articles [16,20,25] reported the time for premature infants to regain birth weight (Figure 3D), and meta-analysis results showed that formula significantly reduced the time for premature infants to regain birth weight (MD = 6.60, 95% CI = 6.11 to 7.08, *p* < 0.00001). A sensitivity analysis showed that heterogeneity remained constant.

#### 3.3.5. Time to Full Enteral Feed

Two articles [14,20] reported the duration of total enteral feeding for premature infants (Figure 3E), and the meta-analysis results showed that donated breast milk reduced the duration of total parenteral feeding for premature infants (MD = −0.33, 95% CI = −3.23 to 2.57, *p* = 0.0002) with low heterogeneity (*I*^2^ = 0%, *p* = 0.69).

### 3.4. Assessment of Risk of Bias

The visual inspection of funnel plots showed no obvious publication bias of included studies (Figures S1 and S2). Figure S3 is the quality evaluation chart of the included papers. Five papers [16,19,22,24,25] did not describe the specific method of double blindness, and two articles [20,23] did not use double blindness, therefore both types were rated as high risk. Four articles [15,19,22,25] did not describe the method of allocation concealment, but the remainders were distributed by hidden opaque envelopes or features of hidden bottles. Four articles [14,19,22,24] did not describe the method of random grouping, which was evaluated as unclear. In the remaining studies, two articles [20,25] were grouped by random number table, and five articles were randomly grouped by a computer. In addition, one article [21] reported incomplete outcome data and was rated as high risk.

## 4. Discussion

Premature birth is a common cause of neonatal mortality. Due to early birth, the fetus body is immature, and various organs cannot function normally, resulting in increased morbidity and mortality. Preterm infants need more nutritional support than for full term newborns to promote growth and development of various parts of the body and formula is commonly used as a way to supply nutrients. In the past decades, however, donated breast milk has become widely accepted as the most reliable nutritional supplement, making donated breast milk an alternative for preterm infants when breast milk is scarce. Unlike formula, donated breast milk retains the immune components and active substances found in fresh breast milk, which promotes the development of bodies and organs of preterm infants, thereby reducing their morbidity and mortality. In this study, a total of 11 randomized controlled trials were included for meta-analysis to establish whether donated breast milk was nutritionally superior to formula for premature infants, especially in terms of body growth, and whether it prevented morbidity and mortality.

The comparison was made using 10 independent outcome indicators including premature infants’ incidence of NEC, length of hospital stay, mortality, incidence of sepsis, duration of parenteral nutrition, and premature retinopathy. In addition, weight gain, head growth, linear growth, the time to regain birth weight, and time to reach to enteral feeding were also assessed. Donated breast milk contains more immune substances, which can enhance the immunity of premature infants. Due to the immature gastrointestinal tract, preterm infants are more predisposed to suffer from NEC in comparison with full term infants. This seriously affects the physical recovery of premature infants and increases their mortality. The results showed that donated breast milk contributed to reducing the incidence of NEC, which was consistent with the meta-analysis results of Yu et al. [12] and Quigley et al. [6]. Breast milk contains substances such as oligosaccharides, white blood cells, cytokines and growth factors that have been shown to reduce inflammation [26]. In addition, SIgA in breast milk provides adequate protection to the gastrointestinal tracts of premature infants by inhibiting pathogen attachment to mucosal surfaces, neutralizing microbial toxins and providing passive immunity [27].

In order to accelerate the physical recovery of premature infants, parenteral nutrition is also used clinically to increase the nutrition of premature infants and promote growth and immunity. Research results show that donated breast milk significantly reduces the time of parenteral nutrition, which means that donated breast milk can promote the growth of the digestive tract more quickly to adapt exclusive oral feeding. Premature infants may suffer from feeding intolerance due to immature organ development. The time to full enteral feed is an important indicator of the physical development of premature infants. The results showed that there was no significant difference between donated breast milk and formula, which was consistent with the results of Quigley et al. [6].

The results showed that formula had significant advantages in weight gain, head circumference and body length growth of premature infants, which was consistent with the meta-analysis results of Quigley et al. [6], although another article [12] reported that formula had no significant advantages in head growth. The possible explanation is that Quigley et al. [6] included more studies, two [19,25] of which showed that formula had a significant advantage in head growth. Formula is more beneficial to the growth of premature infants’ bodies than donated breast milk, possibly because the protein content of formula is higher than that of donated breast milk, and the protein content of general breast milk may not meet the nutritional needs of premature infants [28]. Newborns usually lose body weight after birth, and premature infants are no exception. Premature infants are frail and may take longer to regain their birth weight, which can be increased by adequate nutrition. The conclusion that formula reduced the time to return to birth weight is consistent with Quigley et al.’s [6] study and three RCTs [16,20,25]. This index also shows that the nutrition of formula is more conducive to the physical development of premature infants.

Adequate nutrition increases the rate of development of premature infants’ bodies, leading to shorter hospital stays. The results showed no difference between donated breast milk and formula in this indicator, unlike the results of Yu et al. [12], which included fewer original articles, and none of them used fortifiers to enhance the nutritional content of donated milk, whereas two [14,17] articles that we analyzed used fortifiers and another paper [14] reported that donated milk significantly reduced the length of hospital stay. It becomes obvious that rich nutrients can reduce the length of hospital stay for premature infants. Our results show no difference in premature infant mortality between donated breast milk and formula, which is consistent with the results of the two articles [6,12]. There was no significant difference between donated breast milk and formula in the incidence of sepsis.

The results show that formula is superior to donated breast milk in nutritional composition, but lacks important cytokines and immune components in donated breast milk. Breast-feeding is widely accepted as the preferred method for premature infants, and donated breast milk can play an important role, even though certain nutrients may get lost in storage over time. More research is needed to determine whether the efficacy of donated breast milk is reduced with longer storage and to improve the rational management of the banks of donated breast milk. Before the donated milk can be given to infants, pasteurization is an important step for microbiological purity and safety. Holder pasteurization (HoP) is widely used in human milk banks. This traditional pasteurization method consists of heating the milk to 62.5 °C for 30 min, which may inactivate antibodies and cellular components in the breast milk, such as B cells, T cells, and neutrophils [29]. These bioactive compounds can enhance the immune function of premature infants. Pasteurization also reduces the concentration of the SIgA in donated breast milk [30], so the milk loses some active components. Recently, high temperature short time treatment at 72 °C for 15 s was applied to donated milk processing [31], which functions better in preserving bioactive proteins than HoP. Another novel method, non-thermal high-pressure processing [32], can lead to the preservation of adipokines, growth factor, lactoferrin and IgG much better than or comparable with HoP. It remains an important issue for future study to develop better sterilization methods that can replace traditional pasteurization.

Adding fortifiers to donated breast milk can address the issue of low nutritional content of donated breast milk, and therefore it provides both the nutrition and immunity to maximal effect. In order to make donated milk rich in nutrients, fortifiers may be added to enrich the donated milk with fat and proteins which promote the growth of premature infants. The supplement of fortifiers could compensate for the insufficiency of protein, carbohydrate and minerals in donated milk to promote the physical growth of preterm infants. Our results of subgroup analysis showed that feeding with donated milk containing fortifiers was on a par with formula in head circumference growth. The optimal choice of fortifiers remains to be solved. Another possibility to consider is to combine donated breast milk with formula, but it is unknown whether this will lead to excess nutrition or the physical intolerance of premature infants.

There are limitations to our paper, which are as follows. First, some indicators were only included in two articles, so there might be deviations in the results. Secondly, some articles did not clearly describe randomization and blindness, which might make the reports biased.

## 5. Conclusions

In conclusion, donated breast milk could significantly reduce the incidence of NEC, the duration of parenteral nutrition, and the time of full enteral feeding. Although donated breast milk is inferior to formula in weight gain and body length growth of premature infants, this could be corrected by adding a fortifier to donated milk.

## Figures and Tables

**Table 1:** Characteristics of the included studies.

First Author	Country	Year	Intervention Methods			Gestational Age, Weeks (Intervention/Control)	Birth Weight, g (Intervention/Control)	Number of Patients (Intervention/Control)	Gender (Male/Female)	JADAD Score	Outcomes
			Breast Milk Type	Sterilisation & Preservation	Control Group						
Corpeleijn	Netherlands	2016	Donated Milk	Pasteurized Holder method, 62.5 °C for 30 min	preterm formula	28.3 ± 2.3/28.6 ± 2.2	1064 ± 80/1076 ± 77	373 (183/190)	196/177	6	Incidence of necrotizing enterocolitis (NEC), sepsis, mortality, Incidence of surgery due to NEC
Costa	Italy	2018	Donated Milk	Refrigerate at 4 °C for 24 h, pasteurized Holder method (+62.5 °C for 30 min)	Premature formula, 3.5 g of protein/100 Kcal formula (Plasmon PreZero, Plasmon, Italy).	30 ± 1.9/30.2/1.7	1365 ± 332/1342 ± 275	70 (35/35)	32/38	4	length of hospital stays, sepsis, mortality, time to regain birthweight
Cristofalo	USA	2013	Donated Milk	Pasteurization	Bovine milk based preterm formula (BOV)	27.7 ± 1.5/27.5 ± 2.4	983 ± 207/996 ± 152	53 (29/24)	23/30	6	length of hospital stays, incidence of NEC, sepsis, mortality, retinopathy of prematurity, weight gain, head growth, Linear growth
O’Connor	Canada	2016	Donated Milk	Pasteurized Holder method, 62.5 °C for 30 min	Premature formula (20 or 24 kcal/oz, with 3.0 g of protein/100 kcal).	27.5 ± 2.4/27.8 ± 2.7	995 ± 273/996 ± 272	363 (181/182)	195/168	7	Incidence of NEC, sepsis, mortality, retinopathy of prematurity, weight gain, head growth, Linear growth
Sullivan	USA	2010	Donated Milk	Pasteurization	Bovine milk–based HMF, the enteral intake was 100 mL/kg/d and preterm formula	27.1 ± 2.3/27.3 ± 2.0	909 ± 193/922 ± 197	140 (71/69)	61/79	6	length of hospital stays, incidence of NEC, sepsis, retinopathy of prematurity, bronchopulmonary dysplasia, weight gain, head growth, Linear growth
Schanler	USA	2005	Donated Milk	Holder pasteurization process (62.5 °C for 30 min), kept in −20 °C	Premature formula (100 kJ/oz; Mead Johnson Nutritional Division, Evansville, IN)	27 ± 2/27 ± 2	947 ± 233/957 ± 267	173 (81/92)	92/81	5	Incidence of NEC, sepsis, mortality, meningitis, weight gain, head growth, Linear growth
Putet	France	1984	Donated Milk	Oral feeding was started within 24 to 48 h with pasteurized	Premature formul, containing medium-chain triglycerides (Pregallia, Gallia, France)	30.5 ± 1.5/29.9 ± 1.5	1318 ± 142/1302 ± 269	12 (6/6)	N/A	2	Weight gain, head growth, Linear growth
Lucas	UK	1984	Donated Milk	N/A	Preterm formula	30.8 ± 3.0/31.6 ± 3.1	1431 ± 325/1371 ± 292	62 (29/33)	N/A	5	Weight gain, head growth, Linear growth
Tyson	USA	1983	Donated Milk	Cryopreservation	Preterm formula	29.4 ± 3.1/29.4 ± 2.4	1238 ± 190/1226 ± 197	76 (34/42)	29/47	4	Weight gain, head growth, Linear growth
Gross	USA	1983	Donated Milk	Pasteurized (Holder method, 62.5 °C for 30 min, refrigerated at −20 °C for four months	Preterm formula, 67 kilocalories per deciliter	N/A	N/A	40 (20/20)	N/A	4	Weight gain, head growth, Linear growth, time to regain birthweight
Davies	UK	1977	Donated Milk	Pasteurized (Holder method, 62.5 °C for 30 min, refrigerated at −20 °C	Preterm formula	30.8 ± 0.35/30.4 ± 0.45	1680 ± 0.11/1689 ± 0.11	28 (14/14)	N/A	4	Weight gain, head growth, Linear growth

**Table 2:** Subgroup analysis of length of hospital stay, incidence of NEC, sepsis and mortality.

**Subgroups**	**Length of Hospital Stay**							**Incidence of NEC**						
	**Studies, n**	**Participants, n**	***I* ^2^**	**Q-Test**	**Mean Difference**	**95% CI**	***p***	**Studies, n**	**Participants, n**	***I* ^2^**	**Q-Test**	**Mean Difference**	**95% CI**	***p***
Fortifiers														
YES	2	210	0	0.93	1.45	−1.18, 4.07	0.28	2	539	0	0.32	0.87	0.51, 1.49	0.60
NO	2	416	83	0.01	−4.01	−9.44, 1.42	0.15	3	563	59	0.09	0.56	0.37, 0.86	0.01
Number of participants														
Single	2	276	0.00	0.63	1.63	−0.99, 4.24	0.22							
Multiple	2	123	77.00	0.04	−5.08	−10.63, 0.48	0.07							
**Subgroups**	**Incidence of sepsis**							**Mortality**						
	**Studies, n**	**Participants, n**	***I* ^2^**	**Q-test**	**Mean difference**	**95% CI**	***p***	**Studies, n**	**Participants, n**	***I* ^2^**	**Q-test**	**Mean difference**	**95% CI**	***p***
Fortifiers														
YES	4	955	58	0.07	1.01	0.83, 1.23	0.93							
NO	2	210	0	0.35	1.30	0.71, 2.39	0.39							
Birth weight														
1000–1500 g	2	443	6	0.30	1.15	0.77, 1.73	0.50	2	443	0	0.55	1.17	0.70, 1.97	0.56
<1000 g	4	722	58	0.07	0.99	0.68, 1.44	0.94	3	582	0	0.53	0.81	0.47, 1.41	0.49
Countries														
European	2	443	6	0.30	1.15	0.77, 1.73	0.50	2	443	0	0.55	1.17	0.70, 1.97	0.56
USA	3	359	49	0.14	0.64	0.36, 1.16	0.14	2	219	21	0.26	0.66	0.18, 2.37	0.52
Number of centers														
Single	3	609	46	0.16	1.02	0.78, 1.32	0.90	3	609	0	0.84	1.16	0.71, 1.91	0.55
Multiple	3	556	63	0.07	1.07	0.81, 1.40	0.65	2	416	10	0.29	0.77	0.43, 1.39	0.39
Number of participants														
>100	4	1042	12	0.33	1.11	0.83, 1.48	0.50	3	902	0	0.79	1.01	0.65, 1.56	0.97
<100	2	123	75	0.05	0.70	0.28, 1.74	0.44	2	123	43	0.19	0.60	0.10, 3.56	0.57

**Table 3:** Subgroup analysis of weight gain and head growth.

Subgroups	Weight Gain							Head Growth						
	Studies, n	Participants, n	*I* ^2^	Q-Test	Mean Difference	95% CI	*p*	Studies, n	Participants, n	*I* ^2^	Q-Test	Mean Difference	95% CI	*p*
Fortifiers														
YES	3	545	0	0.60	−2.37	−3.65, −1.09	0.0003	3	545	0	0.42	−0.03	−0.09, 0.03	0.3
NO	6	507	100	<0.00001	−3.49	−3.73, −3.24	<0.00001	6	507	96	<0.00001	−0.07	−0.09, −0.06	<0.00001
Birth weight														
1000–1500 g	3	259	92	<0.00001	−4.47	−5.55, −3.39	<0.00001	3	259	58	0.17	−0.27	−0.37, −0.17	<0.00001
<1000 g	4	685	51	0.11	−0.93	−1.21, −0.65	<0.00001	4	685	0	0.44	0.00	−0.02, 0.01	0.75
Countries														
European	3	253	78	0.003	−3.04	−4.08, −1.99	<0.00001	3	225	30	0.23	−0.10	−0.17, −0.03	0.007
USA	5	473	100	<0.00001	−3.49	−3.74, −3.25	<0.00001	5	475	97	<0.00001	−0.07	−0.09, −0.06	<0.00001
Number of centers														
Single	4	348	98	<0.00001	−10.31	−10.78, −9.85	<0.00001	4	348	83	0.0005	−0.18	−0.20, −0.15	<0.00001
Multiple	4	692	74	0.004	−0.98	−1.26, −0.71	<0.00001	4	692	63	0.03	−0.01	−0.02, 0.01	0.55
Number of participants														
>100	4	685	51	0.11	−0.93	−1.21, −0.65	<0.00001	4	685	0	0.44	0.00	−0.02, 0.01	0.75
<100	5	367	98	<0.00001	−9.75	−10.19, −9.31	<0.00001	5	341	72	0.003	−0.18	−0.20, −0.15	<0.00001
Date of publication														
<2010	6	533	98	<0.00001	−9.36	−9.79, −8.93	<0.00001	6	507	70	0.003	−0.18	−0.20, −0.15	<0.00001
≥2010	3	519	0	0.66	−0.88	−1.16, −0.60	<0.00001	3	519	26	0.75	0.00	−0.02, 0.01	0.75

**Table 4:** Subgroup analysis of head growth.

Subgroups	Body Length Growth						
	Studies, n	Participants, n	*I* ^2^	Q-Test	Mean Difference	95% CI	*p*
Fortifiers							
YES	3	545	79	0.01	−0.18	−0.30, −0.06	0.003
NO	6	507	89	<0.00001	−0.13	−0.14, −0.11	<0.00001
Birth weight							
1000–1500 g	3	259	60	0.06	-0.21	-0.29,-0.13	<0.00001
<1000 g	4	685	78	0.004	−0.07	−0.09, −0.04	<0.00001
Countries							
European	3	151	48	0.13	−0.14	−0.20, −0.07	<0.00001
USA	5	475	93	<0.00001	−0.13	−0.15, −0.11	<0.00001
Number of centers							
Single	4	350	80	0.0020	−0.17	−0.20, −0.15	<0.00001
Multiple	4	590	71	0.01	−0.07	−0.10, −0.05	<0.00001
Number of participants							
>100	4	685	78	0.004	−0.07	−0.09, −0.04	<0.00001
<100	5	267	57	0.040	−0.18	−0.20, −0.15	<0.00001
Date of publication							
<2010	6	433	68	0.004	−0.18	−0.20, −0.15	<0.00001
≥2010	3	522	79	0.008	−0.07	−0.09, −0.04	<0.00001

## Data Availability

Data will be provided upon request.

## Supplementary Materials

The following supporting information can be downloaded at: https://www.mdpi.com/article/10.3390/nu14091724/s1, Figure S1. The funnel plots of primary outcomes. (A) Incidence of NEC (B) Length of Hospital Stay (C) Mortality (D) Incidence of sepsis (E) Duration of parenteral nutrition (F)Incidence of retinopathy of prematurity. Figure S2. The funnel plots of secondary outcomes. (A) Weight gain (B) Head growth (C) Linear growth (D) Time to regain birth weight (E) Time to full enteral feeding. Figure S3. RCT risk of bias summary for included Randomized Controlled Trial.
