# Supplementary material for: Efficacy of Donated Milk in Early Nutrition of Preterm Infants: A Meta-Analysis
Source: Nutrients. 2022 Apr 21;14(9):1724. doi: 10.3390/nu14091724 (PMC9105142; doi:10.3390/nu14091724)
Supplement: Supplementary file 1 [file nutrients-14-01724-s001.zip › nutrients-1669021-supplementary.pdf]

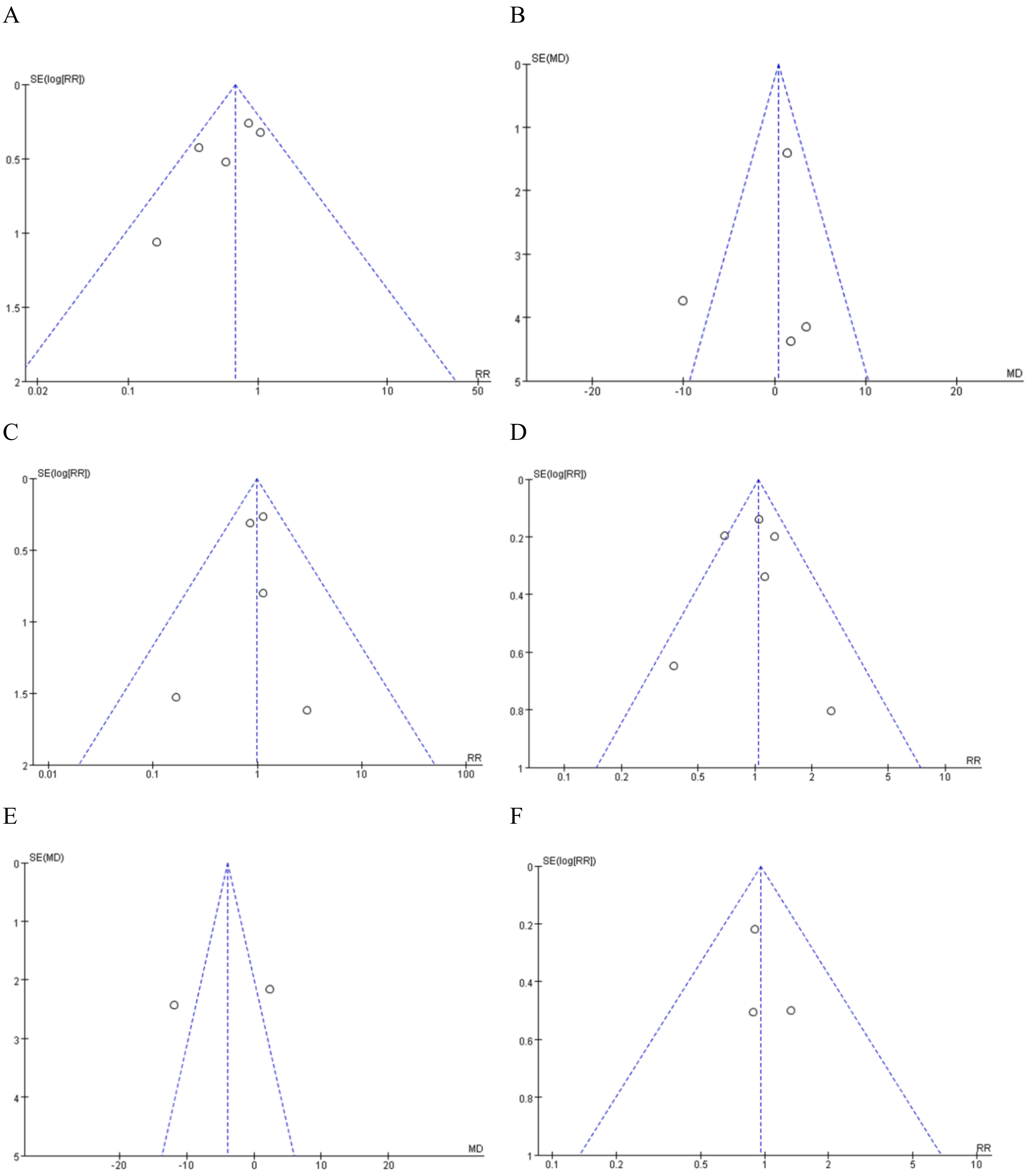


Figure S1. The funnel plots of primary outcomes. (A) Incidence of NEC (B) Length of Hospital Stay (C) Mortality (D) Incidence of sepsis (E) Duration of parenteral nutrition (F)Incidence of retinopathy of prematurity.

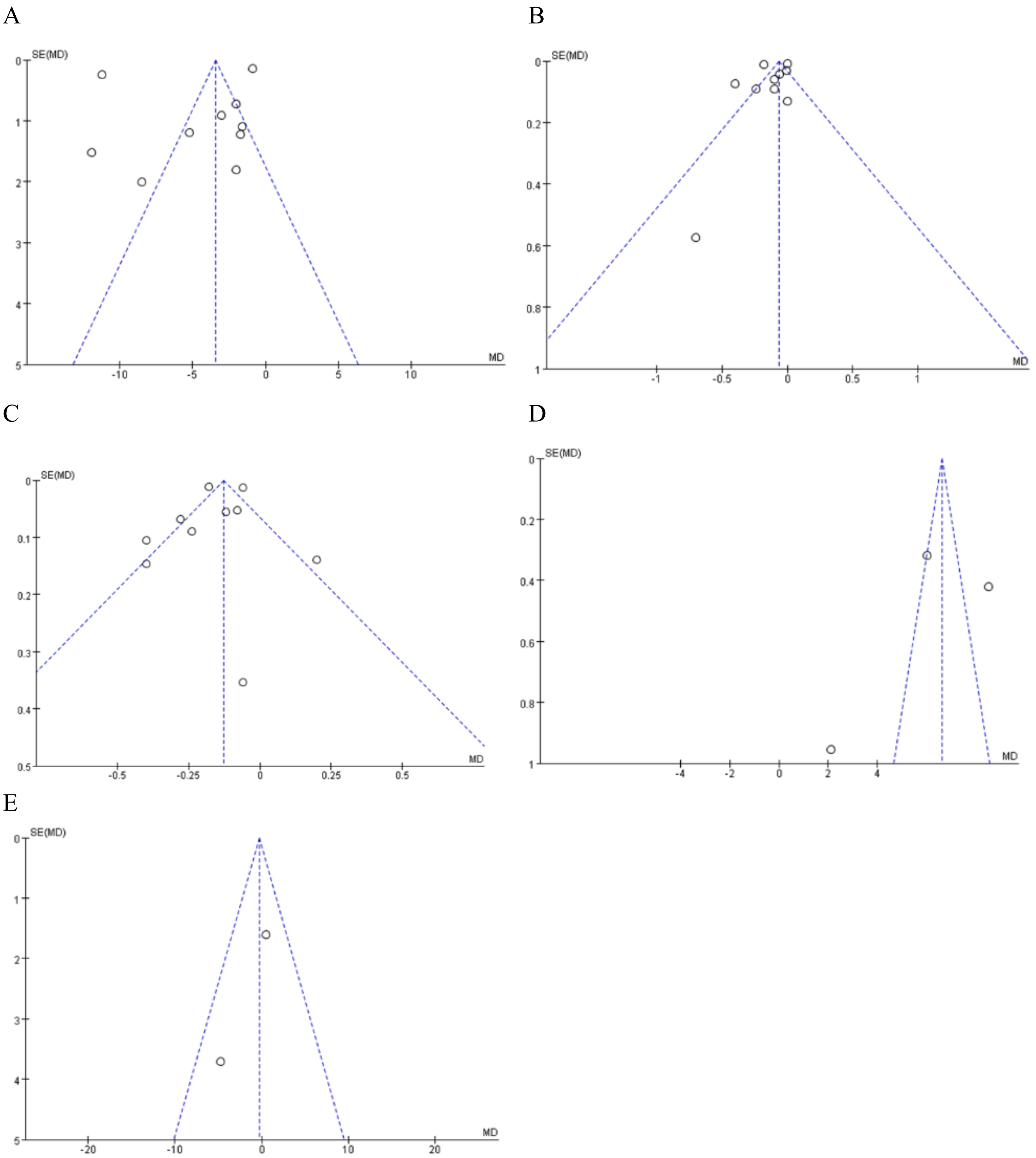


Figure S2. The funnel plots of secondary outcomes. (A) Weight gain (B) Head growth (C) Linear growth (D) Time to regain birth weight (E) Time to full enteral feeding.

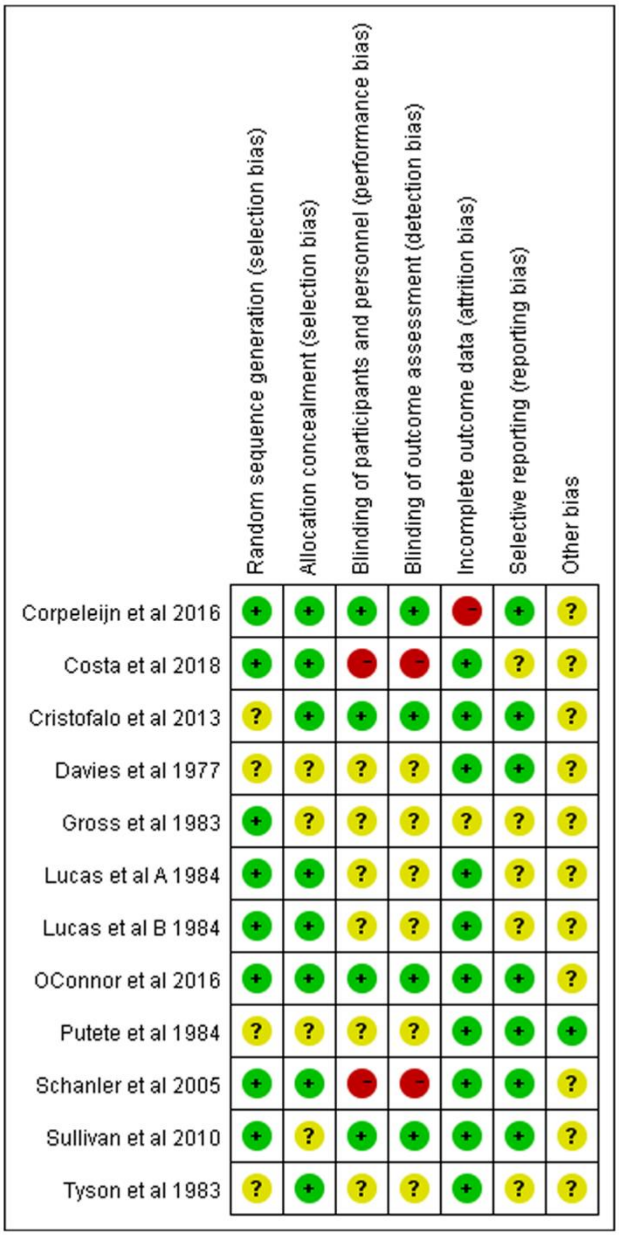


Figure S3. RCT risk of bias summary for included Randomized Controlled Trial.
